# Contact X-ray Brachytherapy as a sole treatment in selected patients with early rectal cancer – Multi-centre study

**DOI:** 10.1016/j.ctro.2024.100851

**Published:** 2024-09-06

**Authors:** Ngu Wah Than, D. Mark Pritchard, David M. Hughes, Kai Shing Yu, Helen S. Minnaar, Amandeep Dhadda, Jamie Mills, Joakim Folkesson, Calin Radu, C.A. Duckworth, Helen Wong, Muneeb Ul Haq, Rajaram Sripadam, Mark D. Halling-Brown, Alexandra J. Stewart, Arthur Sun Myint

**Affiliations:** a Department of Molecular and Clinical Cancer Medicine, Institute of Systems, Molecular and Integrative Biology, The University of Liverpool, L69 3GE, UK; b The Clatterbridge Cancer Centre NHS Foundation Trust, 65 Pembroke Place, Liverpool L7 8YA, UK; c Department of Health Data Science, Institute of Population Health, The University of Liverpool, L7 3EA, UK; d St. Luke’s Cancer Centre, Royal Surrey Hospital, Guildford, Surrey, UK; e Castle Hill Hospital, Castle Road, Cottingham HU16 5JQ, UK; f Nottingham University Hospitals NHS Trust, Nottingham City Hospital, Hucknall Rd, Nottingham NG5 1PB, UK; g Department of Surgery, Akademiska sjukhuset, Institute of Surgical Sciences, Uppsala University, 751 85 Uppsala, Sweden; h Department of Immunology, Genetics and Pathology, Experimental and Clinical Oncology, Uppsala University, 751 85 Uppsala, Sweden; i University of Surrey, Guildford, Surrey, UK

**Keywords:** Rectal cancer, Contact X-ray Brachytherapy, Papillon, Organ preservation, Sole treatment

## Abstract

**Background and purpose:**

Radical surgery is the standard of care for early rectal cancer. However, alternative organ-preserving approaches are attractive, especially in frail or elderly patients as these avoid surgical complications. We have assessed the efficacy of sole Contact X-ray Brachytherapy (CXB) treatment in stage-1 rectal cancer patients who were unsuitable for or declined surgery.

**Materials and methods:**

This retrospective multi-centre study (2009–2021) evaluated 76 patients with T1/2-N0-M0 rectal adenocarcinomas who were treated with CXB alone. Outcomes were assessed for the entire cohort and sub-groups based on the T-stage and the criteria for receiving CXB alone; Group A: patients who were fit enough for surgery but declined, Group B: patients who were high-risk for surgery and Group C: patients who had received prior pelvic radiation for a different cancer.

**Results:**

With a median follow-up of 26(IQR:12–49) months, initial clinical Complete Response (cCR) was 82(70–93)% with rates of local regrowth 18(8–29)%, 3-year actuarial local control (LC) 84(75–95)%, distant relapse 3 %, and no nodal relapse. 5-year disease-free survival (DFS) and overall survival (OS) were 66(48–78)% and 58(44–75)%. Lower OS was observed in Groups B [HR:2.54(95 %CI:1.17, 5.59), p = 0.02] and C [HR:2.75(95 %CI:1.15, 6.58), p = 0.03]. Previous pelvic radiation predicted lower cCR and OS. The main toxicity was G1-2 rectal bleeding (26 %) and symptoms of impaired anal sphincter function were not reported in any patients.

**Conclusion:**

CXB treatment alone achieved a high cCR rate with satisfactory LC and DFS. Inferior oncological outcomes were observed in patients who had received prior pelvic radiotherapy. CXB alone, with its favourable toxicity profile and avoidance of general anaesthesia and surgery risks, therefore, can be considered for patients who are unsuitable for or refuse surgery.

## Highlights

• We have evaluated the role of sole Contact X-ray brachytherapy (CXB) as a treatment option for early rectal cancer.
• There have been no recently published analyses of the efficacy of CXB as a sole modality treatment for early rectal cancer.
• A high clinical complete response rate (82%), low local regrowth rate (18%), and few distant tumour relapses were observed.
• We observed poorer outcomes in patients who had received prior pelvic radiotherapy for other cancers.

## Introduction

The standard management of early rectal cancer (T1/T2-N0-M0) involves total mesorectal excision (TME) [1], [2]. This modality offers an impressive cure rate (90–94 %) and 5-year survival rates exceeding 90 % [3]. However, it is associated with increased risks of postoperative complications and impacts upon long-term functional outcomes [4], [5]. Especially in patients with advancing age and those with multiple comorbidities [6].

Over the last decade, increased detection of early-stage rectal cancers through national bowel cancer screening programmes [7], [8], a higher incidence of bowel cancer in the elderly [9], and the increased prevalence of medical comorbidities associated with advancing age [10], support the use of organ-preserving treatments. These include transanal excision (TAE) or endoscopic mucosal resection (EMR) with or without adjuvant radiotherapy, and non-surgical treatment modalities including external beam (chemo)radiation (EBRT/EBCRT), or exclusive endocavitary radiotherapy [11], [12], [13], [14], [15], [16]. Adopting these more conservative strategies aims to minimise post-surgical complications, decrease mortality rates, and enhance patients’ overall quality of life [17], [18].

Since its introduction as a rectal cancer treatment, Contact X-ray Brachytherapy (CXB) has been used as a standalone therapy for very early-stage tumours and can be supplemented by the incorporation of EBRT or interstitial brachytherapy using Iridium (^192^Ir) [19], [20]. Sole CXB treatment remains an attractive option for early-stage rectal cancers in view of the low likelihood of lymphatic metastasis in T1/T2 tumours (12–22 %) [21], [22].

Appelt et al. [23] demonstrated a clear dose–response relationship in patients with locally advanced rectal cancer who underwent neoadjuvant chemoradiation. The therapeutic efficacy of radiation relies on balancing tumour control and minimising side effects in normal tissues. Despite advanced precision in external radiotherapy techniques, achieving accurate tumour irradiation without damaging nearby healthy tissues remains challenging [24]. CXB has the advantage of delivering a high radiation dose directly to the tumour (20–30 Gy/session), with a rapid dose fall-off as tissue depth increases due to its low energy (50kVp), (50 % of the dose at 5 mm depth and 30 % at 10 mm depth), thereby minimising damage to the surrounding normal tissues [25], [26].

Although the oncological outcomes following CXB alone may not be comparable to the standard surgical results [27], it offers significant advantages for certain patients, particularly those who have limited capacity to undergo surgical procedures. CXB is an ambulatory treatment that generally does not require general anaesthesia, making it suitable for patients who are at high surgical risk due to advanced age or multiple comorbidities. Additionally, patients can resume their daily activities soon after treatment [26], [27]. These result in lower overall treatment costs for this therapy compared to major surgical procedures [28]. Furthermore, CXB treatment due to its limited penetration properties, avoids disruption of the surgical *meso*-rectal plane. This is an important advantage over local excision, which can impact post-surgical morbidities if subsequent salvage completion surgery is required following local treatment failure [29], [30].

Although numerous studies over the past seven decades have examined the outcomes of CXB alone, there has been a lack of recent multi-centre reports with meaningful numbers of patients. We therefore conducted this multi-institutional study to evaluate the efficacy of sole CXB treatment with curative intent, particularly in the older population who were considered high surgical risk or those who declined surgery for various reasons. We also assessed the prognostic factors influencing oncological outcomes and the tolerability of treatment.

## Materials and methods

### Patient selection

This retrospective multi-centre study utilised data from the Guildford Colorectal Database System, a prospectively maintained national database employed by four UK CXB treatment centres: St. Luke's Cancer Centre (Guildford), Clatterbridge Cancer Centre (Liverpool), Nottingham City Hospital, and Queen's Oncology Centre (Hull), as well as Uppsala Cancer Unit in Sweden. The study included all consecutive patients with well/moderately differentiated, (cT1-2,N0,M0) rectal adenocarcinoma who received CXB treatment alone with curative intent between 2009 and 2021. Patients with more advanced tumour stages (cT3/T4,N1, or M1) were excluded, as CXB alone is unlikely to cure such cases due to its limited penetration into deeper tissues. We also excluded patients who received CXB alone as adjuvant treatment after local excision or for luminal regrowth during watch-and-wait after neoadjuvant (chemo)radiation, as these cases have been analysed separately [12], [31]. After obtaining approval from all participating institutions, we collected data on patient and tumour characteristics/staging, treatment details, and follow-up information. For missing data not recorded in the system, we retrieved information where possible from local patient records.

### Patient assessment and CXB treatment

Baseline tumour characteristics and staging were evaluated through clinical examination, endoscopic assessment, histological confirmation, cross-sectional imaging (magnetic resonance imaging (MRI)), whichwas mainly used for determining tumour and nodal stage unless it was contraindicated (e.g. patient with a cardiac pacemaker) and computed tomography (CT) (chest and abdomen) to exclude any distant metastases. FDG-PET/CT was not routinely performed unless it was needed to evaluate an equivocal finding on a contrast-enhanced CT or MRI scan or in patients who had strong contraindications to IV contrast administration.

Before starting CXB treatment, patients were reviewed at the local CXB multi-disciplinary team (MDT) meeting to determine the suitability and intent of treatment. Each patient was counselled to fully understand that CXB alone is not a standard of care for early-stage rectal cancer and that they may need to consider surgery if there is residual tumour or local regrowth after CXB. All patients provided informed consent before treatment commencement.

CXB was delivered as an outpatient treatment using the Papillon-50 ©machine (50kVp X-rays (HVL 0.64 Al, 2.7 mA), Ariane, Alfreton, UK), 20–30 Gy per fraction delivered every 2 weeks apart, through a rectal treatment applicator (size 30, 25, or 22 mm) at a focal source surface distance of 29, 32, or 38 mm respectively. A standard dose of 90 Gy (rectal mucosal surface dose) was delivered in 3 fractions over 4 weeks, with a fourth 20 Gy dose (total 110 Gy) being administered to selected patients who had minimal visible and/ palpable tumours still present after the third fraction.

### Follow-up

Patients who achieved a complete/near Clinical Response (cCR/nCR) at 8–12 weeks following completion of treatment underwent regular assessments, including digital rectal examination, flexible sigmoidoscopy or rectoscopy at their local centre and CXB centre alternatively, and MRI scans at subsequent 12–14 week intervals during the first 2 years where the likelihood of recurrence is highest. Subsequently, evaluations were conducted every 6 months in the 3rd year. Usually, only endoscopic examinations were performed in the 4th and 5th years. Patients who did not achieve cCR/nCR, which was confirmed by triple assessment (DRE, endoscopy, and MRI) with/without histological confirmation, by 24 weeks were considered for surgical treatment options if they were agreeable and fit enough.

### Outcome measures

Oncological outcomes were evaluated for the whole group and sub-groups based on tumour stage (T1 versus T2) and the reasons for undergoing sole CXB without involving EBRT. The patients were divided into 3 groups as follows; those patients who were fit enough for surgery but refused (group A), those who had a high risk for surgery due to age and/or medical comorbidities (group B), and those who had a history of prior pelvic radiotherapy for other malignancies including prostate (n = 13), gynaecological (n = 6), and bladder (n = 1) cancers (group C). The primary endpoints included cCR, local control rate (LCR), disease-free survival (DFS), and overall survival (OS). Disease-free survival for patients who have achieved cCR was calculated from the date of the last radiotherapy treatment to the date of locoregional recurrence after R0/R1 resection of the primary tumour, non-salvageable local regrowth/R2 salvage resection, the occurrence of a second primary, distant metastasis, or last follow-up. For those who had undergone salvage surgery for their residual disease, disease-free survival was from the date of R0/R1 resection of the primary tumour to the date of locoregional recurrence, the occurrence of a second primary, distant metastasis, or last follow-up. Overall survival was defined as the period from the date of diagnosis to the date of the last data review or death from any cause. Secondary outcomes involved assessing the influence of patient and tumour factors on oncological outcomes and post-CXB radiation toxicities using Common Terminology Criteria for Adverse Events (CTCAE) criteria version 5.0 [36].

### Statistical analysis

Quantitative data were expressed as medians with interquartile ranges (IQR) or means with standard deviations (SD), while categorical data were reported as counts with percentages. Categorical variables were compared using the χ2 test, with Fisher’s exact test (two-tailed) employed when necessary. Continuous variables, which were not normally distributed, were compared using the independent-samples Mann-Whitney *U* test. Survival differences were examined with Kaplan-Meier curves, and the Log-rank test was used for statistical assessment. Associations between tumour characteristics and survival risks were analysed using Cox proportional hazards models, while logistic regression was used to evaluate binary margin outcomes. A p-value of < 0.05 was considered statistically significant. Statistical analyses were conducted using R version 4.3.2.

## Results

**Fig. 1:**
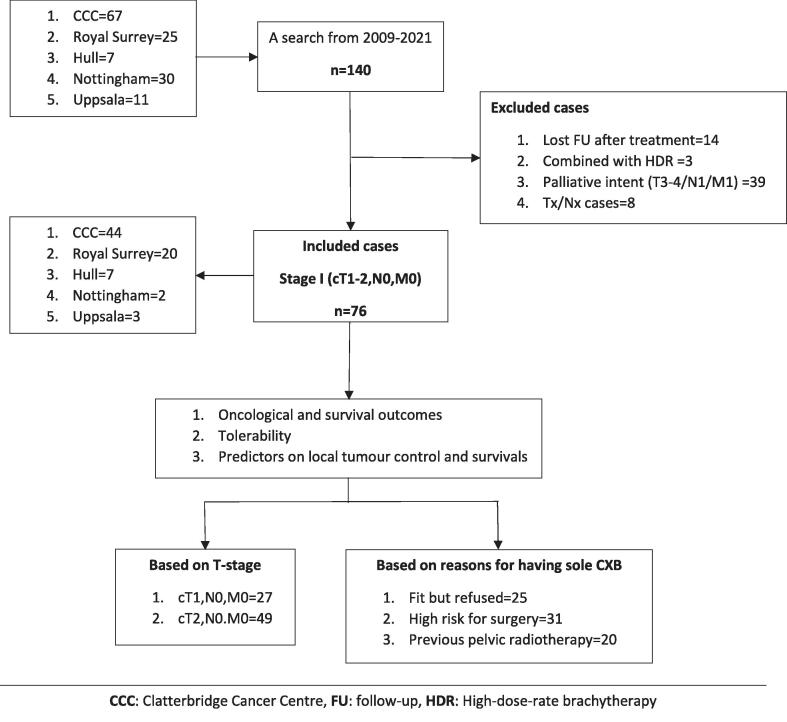
Flowchart of study profile.

From 2009 to 2021, 140 patients in the database were treated solely with CXB. However, only 76 patients were eligible for the study after excluding those who had more advanced stages (cT3-4/N1/M1) (n = 39), individuals not having accurate staging (cTx/Nx/Mx) (n = 8), patients who had been lost to follow-up (n = 14) and those who underwent combined treatment with high-dose-rate brachytherapy (n = 3). Oncological and clinical outcomes were analysed for the entire group and sub-groups based on the tumour stage and the reasons for administering CXB alone. Detailed study characteristics are presented in Fig. 1.

### Whole group analysis

With a median follow-up of 26(IQR: 12–49) months, the median age of patients was 78(IQR: 67–84) years and the majority (82 %) were aged between 65–98 years. Approximately 47 % of patients, despite being apparently fit, declined surgery. The mean tumour size was 2.4 ± 1.0 cm. Demographic data for the whole group and subgroups are shown in Table 1.

**Table 1:** Patient and tumour characteristics of the whole and sub-groups.

Characteristics		Total	Based on reasons for having sole CXB				Based on T-stage		
		n = 76 (%)	Fit	High risk	Previous RT	P value	T1-N0-M0	T2-N0-M0	P value
			n = 25(%)	n = 31(%)	n = 20(%)		n = 27(%)	n = 49(%)	
Median follow-up (IQR) (months)		26(12–49)	43(19–64)	25(12–40)	16(9–41)	0.03	44(14–60)	24(11–42)	0.03^ǂ^
Age (years)	Median (IQR)	78(67–84)	75(65,85)	81(67,86)	77(68–81)	0.21	74(67–82)	80(67–84)	0.54^ǂ^
Age group	48-<65 years	14(18)	7(28)	3(10)	4(20)		4(15)	10(20)	
	≥65-98 years	62(82)	18(72)	28(90)	16(80)		23(85)	39(80)	
Gender	Male	49(65)	17(68)	19(61)	13(65)	0.87	20(74)	29(59)	0.19*
	Female	27(35)	8(32)	12(39)	7(35)		7(26)	20(41)	
WHO PS	0–1	45(59)	22(88)	9(29)	14(70)	<0.001	21(78)	24(49)	0.04*
	02-Mar	27(36)	3(12)	20(65)	4(20)		6(22)	21(43)	
	Not recorded	4(5)		2(6)	2(10)		0(0)	4(8)	
Refused surgery		34(45)	25(100)	3(10)	6(30)	<0.001	13(48)	23(47)	0.66*
Reason having CXB	Fit	28(37)					14(52)	14(29)	0.10*
	High risk	29(38)					9(33)	20(41)	
	Prior pelvic RT	19(25)					4(15)	15(31)	
TNM stage	T1N0M0	27(36)	12(48)	11(35)	4(20)	0.15			
	T2N0M0	49(64)	13(52)	20(65)	16(80)				
Grade	Well	4(5)	3(12)	1(3)	0(0)	0.2	2(7)	2(4)	0.54*
	Moderate	42(55)	16(64)	16(52)	10(50)		16(59)	26(53)	
	Unspecified	18(24)	3(12)	8(26)	7(35)		4(15)	14(29)	
	Not recorded	12(16)	3(12)	6(19)	3(15)		5(19)	7(14)	
Tumour morphology	Exophytic	38(50)	11(44)	16(52)	11(55)	0.58	11(41)	27(55)	0.31*
	Flat/Ulcerated	6(8)	1(4)	2(6)	3(15)		0(0)	6(12)	
	Not recorded	32(42)	13(52)	13(42)	6(30)		16(59)	16(33)	
Tumour size	Mean (cm)	2.4 ± 1.0	2.3 ± 1.0	2.2 ± 0.9	2.8 ± 1.2	0.19	2.3 ± 1.3	2.4 ± 0.9	0.31^ǂ^
Tumour size group	≤3cm	54(71)	17(68)	25(81)	12(60)	0.78	18(67)	36(73)	1.0*
	>3–4.3 cm	6(8)	2(8)	2(6)	2(10)		2(7)	4(6)	
	Not recorded	16(21)	6(24)	4(13)	6(30)		7(26)	9(21)	
Distance from the anal verge	3-≤6cm	48(63)	14(56)	20(65)	14(70)	0.47	20(74)	28(57)	0.04*
	>6–12 cm	20(26)	7(28)	10(32)	3(15)		3(11)	17(35)	
	Not recorded	8(11)	4(16)	1(3)	3(15)		4(15)	4(7)	
CXB total dose	90 Gy	21(28)	8(32)	10(32)	3(15)	0.34	4(15)	17(35)	0.06*
	110–120 Gy	55(72)	17(68)	21(68)	17(85)		23(85)	32(65)	

IQR: Interquartile Range, WHO: World Health Organization, CXB: Contact X-ray Brachytherapy, ǂ= Mann-Whitney *U* test, *= χ^2^ test.

The initial cCR rate was 82 % and local regrowth, defined as a regrowth at the site of the original tumour at which CXB had been delivered, occurred in 18 % of patients within 7–43 (median: 14) months after the last treatment, resulting in an overall 3-year actuarial local control rate of 84 %. The one-, three- and five-year DFS were 80 %(95 %CI:73–95), 70 %(95 %CI:60–82), and 66 %(95 %CI:53–82) respectively and OS were 97 %(95 %CI:94–100), 75 %(95 %CI:70–87), and 58 %(95 %CI:48–72) over the same periods. (Fig. 3A, Fig. 4A) Regional relapse occurred in only two patients (who both had T1 tumours) (3 %), and nodal relapse was not observed in any patients. Two patients (3 %) experienced distant relapses (both had T2 tumours). The comprehensive outcomes with absolute numbers for the whole group and sub-groups are presented in Table 2 and Fig. 2A, Fig. 2B.

**Table 2:** Oncological outcomes of the whole and sub-groups.

Characteristics	Total n = 76 (%)	Based on reasons for having sole CXB				Based on T-stage		
		Fit n = 25(%)	High risk n = 31(%)	Previous RT n = 20(%)	P value (χ^2^ test)	T1-N0-M0 n = 27(%)	T2-N0-M0 n = 49(%)	P value (χ^2^ test)
Clinical complete response	62(82)	23(92)	25(81)	14(70)	0.17	25(93)	37(76)	0.05
Residual disease	14(18)	2(8)	6(19)	6(30)		2(7)	12(24)	
Local regrowth	11/62(18)	5/23(22)	2/25(8)	4/14(29)	0.03	4/25(16)	7/37(19)	0.95
Regional relapse	2(3)	1(4)	0(0)	1(5)	0.36	2(7)	0(0)	0.12
Distant relapse	2(3)	0(0)	1(3)	1(5)	0.38	0(0)	2(4)	0.54

**Fig. 2A:**
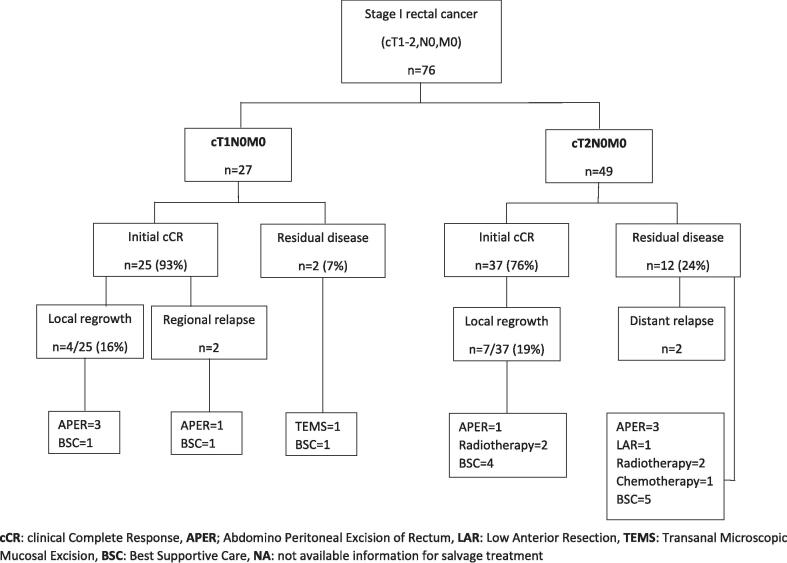
Flow diagram illustrating the oncological outcomes of sub-groups based on T-stage.

**Fig. 2B:**
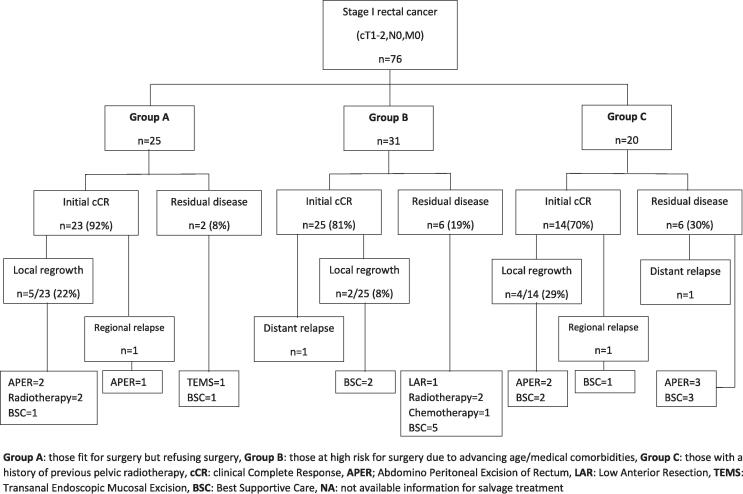
Flow diagram illustrating the oncological outcomes of sub-groups based on reasons for having CXB alone. Group A: those fit for surgery but refusing surgery, Group B: those at high risk for surgery due to advancing age/medical comorbidities, Group C: those with a history of previous pelvic radiotherapy, cCR: clinical Complete Response, APER; Abdomino Peritoneal Excision of Rectum, LAR: Low Anterior Resection, TEMS: Transanal Endoscopic Mucosal Excision, BSC: Best Supportive Care, NA: not available information for salvage treatment.

### Sub-group analyses

#### Based on the T-stage

A total of 76 patients were included: consisting of T1 (n = 27) and T2 (n = 49) patients. The median ages were 74(IQR: 67–82) years and 80(IQR: 67–84) years. The median follow-up periods were 44(IQR: 14–60) months and 24(IQR: 11–42) months respectively.

The cCR rates of T1 and T2 patients were 93 % and 76 % (p = 0.05). Local regrowth rates were not significantly different between groups (16 % vs 19 %, p = 0.95) with 3-year actuarial local control rates of (90 % vs 80 %, p = 0.53). Both DFS and OS did not show statistical differences between T1 and T2 patients with DFS rates of 82 % vs 78 %, 78 % vs 67 %, and 72 % vs 67 % at 1 year, 3 years and 5 years respectively [HR:1.2(95 %CI: 0.4, 3.1), p = 0.58]. Regarding OS, these were 96 % vs 98 %, 85 % vs 70 %, and 70 % vs 52 % at 1 year, 3 years and 5 years [HR:1.6(95 %CI:0.8, 3.2), p = 0.15]. (Fig. 3B, Fig. 4B).

**Fig. 3A:**
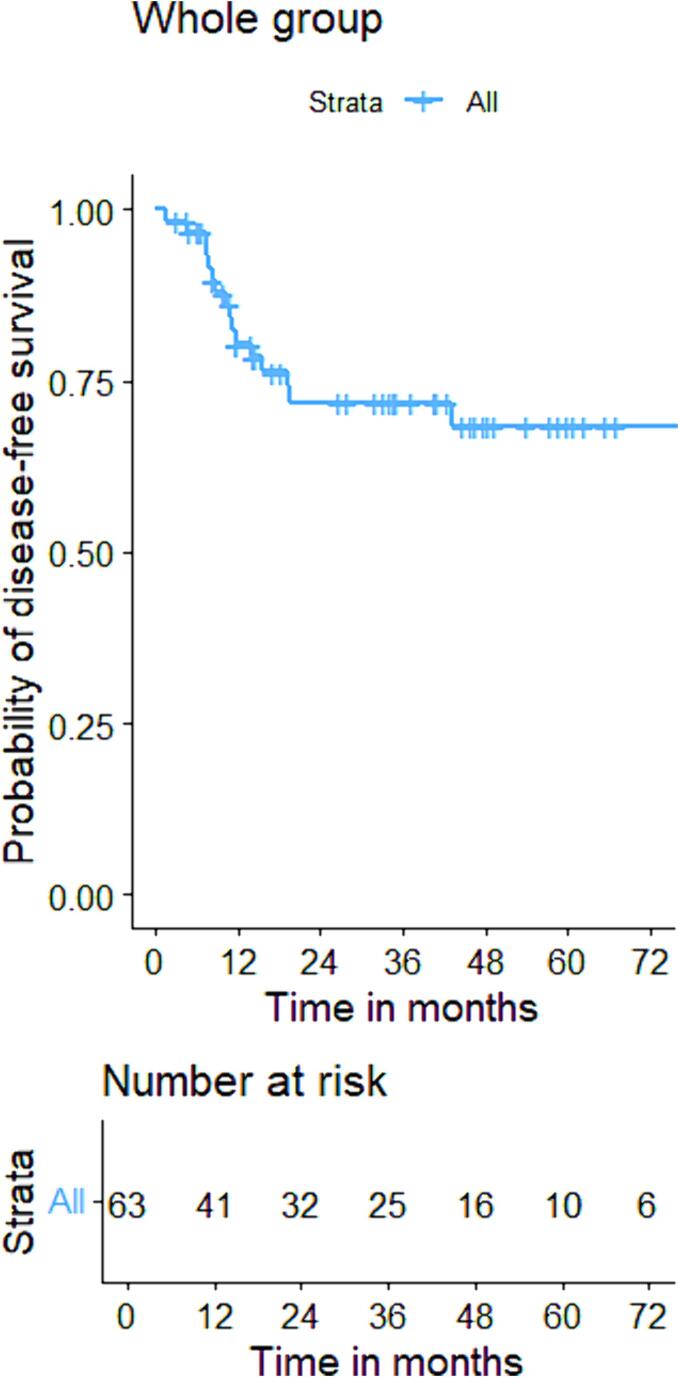
Kaplan-Meier curve for disease-free survival of the whole group.

**Fig. 3B:**
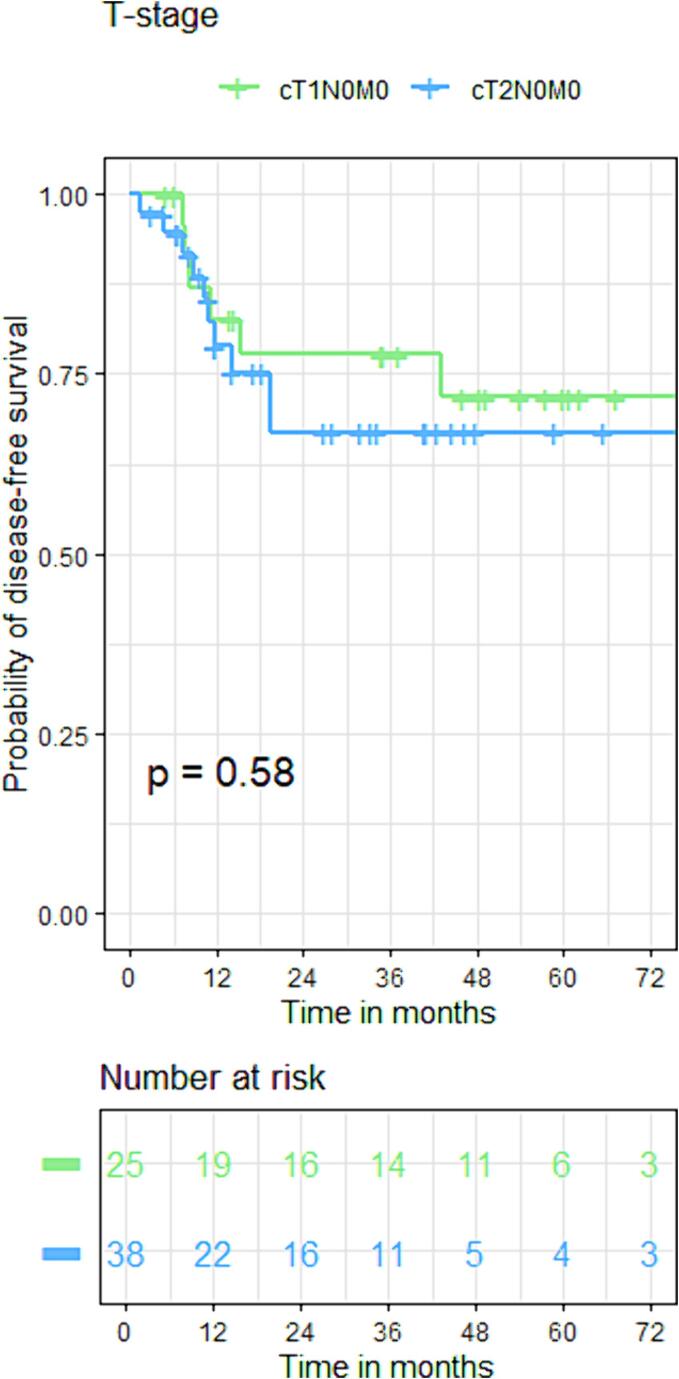
Kaplan-Meier curve for disease-free survival based on T-stage.

#### Based on reasons for having CXB alone

Of the 76 patients, 25 belonged to Group A (fit but refused surgery), 31 to Group B (high risk for surgery), and 20 to Group C (history of prior pelvic radiotherapy). The median follow-up periods were 43[IQR: 19–64] months, 25[IQR: 12–40] months, and 16[IQR: 9–41] months.

The initial cCR rates were 92 %, 81 %, and 70 % (p = 0.17) for groups A, B, and C. Groups A (22 %) and C (29 %) exhibited significantly higher local regrowth rates than group B (8 %) (p = 0.03). The 3-year local control rates for groups A, B, and C were 80 %, 95 %, and 75 % (p = 0.32). DFS was not significantly different across groups: (86 %, 86 %, 60 %) at 1 year; (70 %, 86 %, 48 %) at 3 years; and (65 %,78 %, 48 %) at 5 years for groups A, B, and C [HR: 2.21(95 %CI: 0.70, 6.92), p = 0.180]. However, the OS was significantly lower in groups B [HR:2.54(95 %CI:1.17, 5.59), p = 0.02] and C [HR:2.75(95 %CI:1.15, 6.58), p = 0.03] compared to group A: (100 %, 100 %, 90 %) at 1 year; (97 %, 65 %, 60 %) at 3 years, and (75 %, 55 %, 44 %) at 5 years for group A, B, and C respectively. (Fig. 3C, Fig. 4C).

**Fig. 3C:**
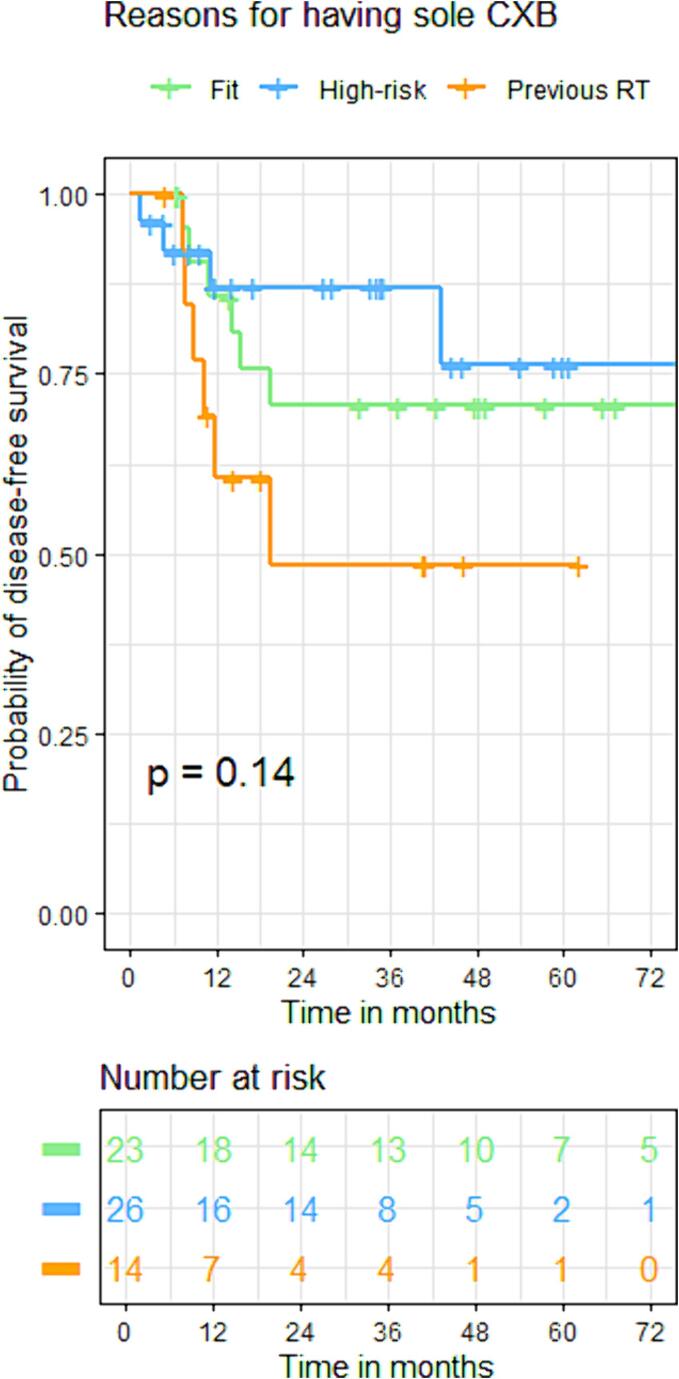
Kaplan-Meier curve for disease-free survival based on reasons for having CXB alone.

**Fig. 4A:**
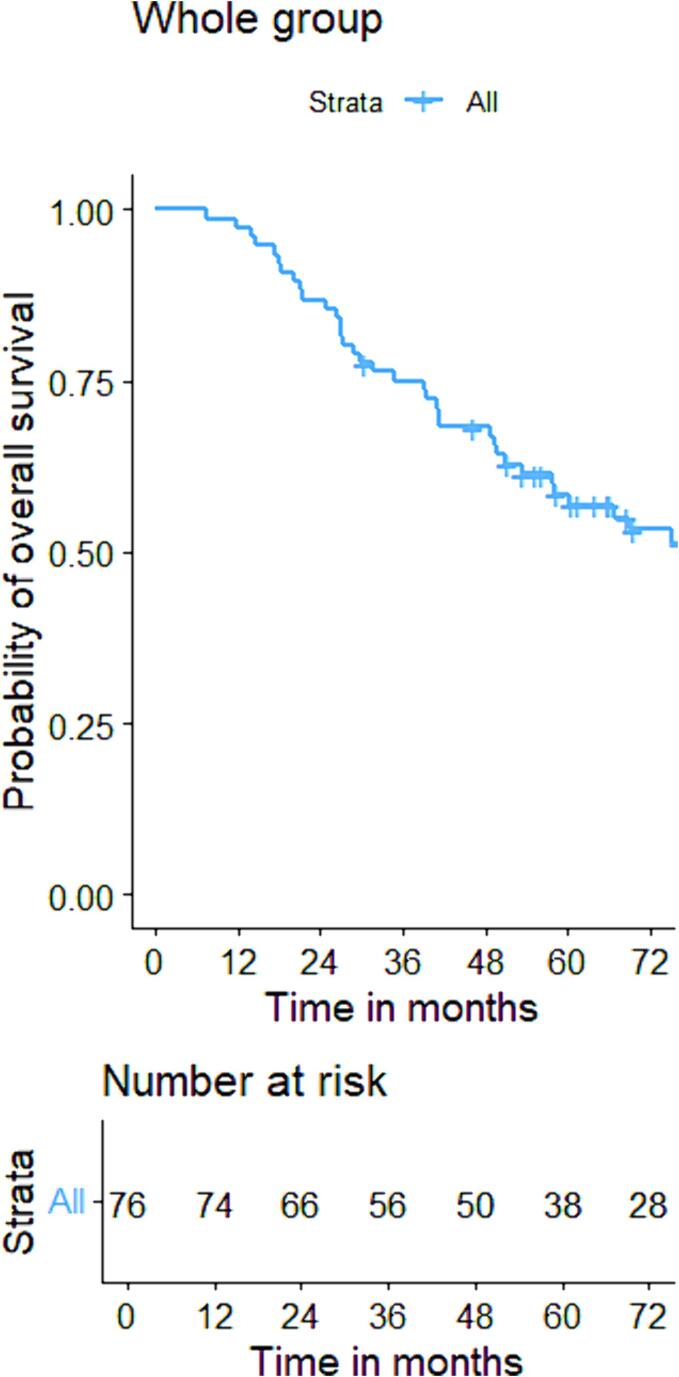
Kaplan-Meier curve for overall survival of the whole group.

**Fig. 4B:**
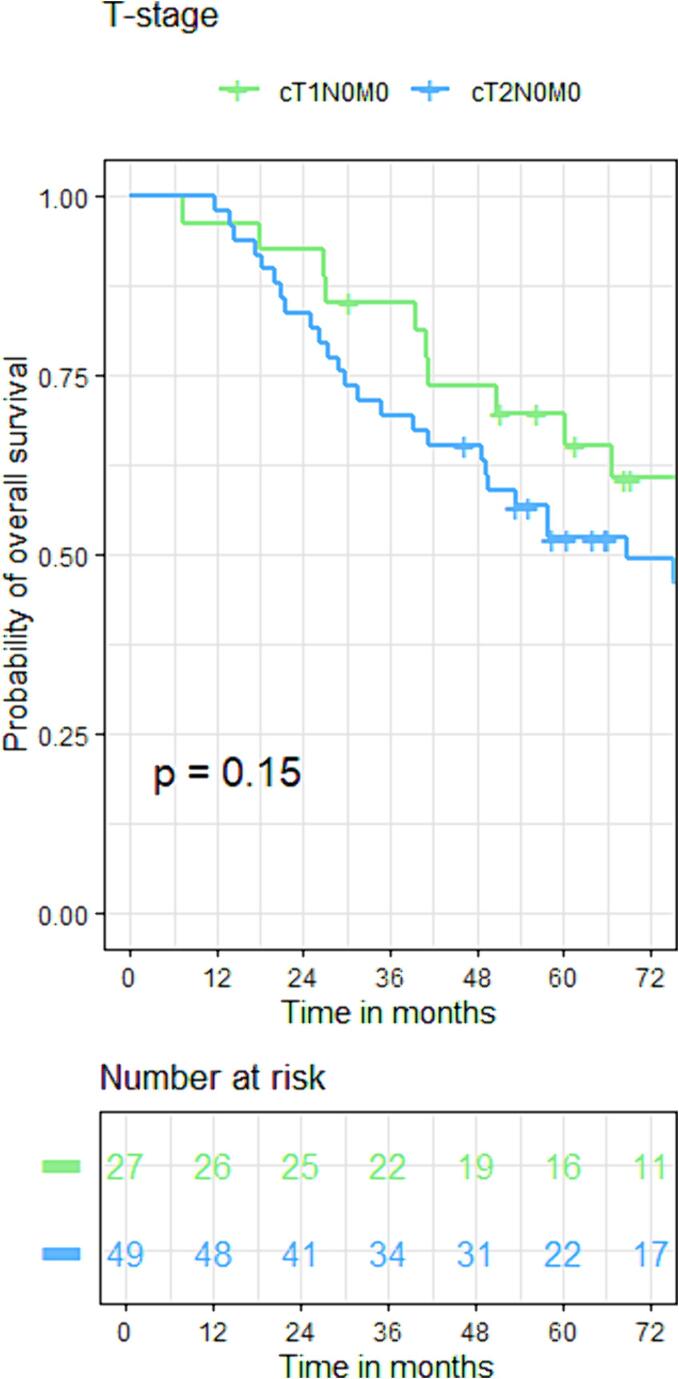
Kaplan-Meier curve for overall survival based on T-stage.

**Fig. 4C:**
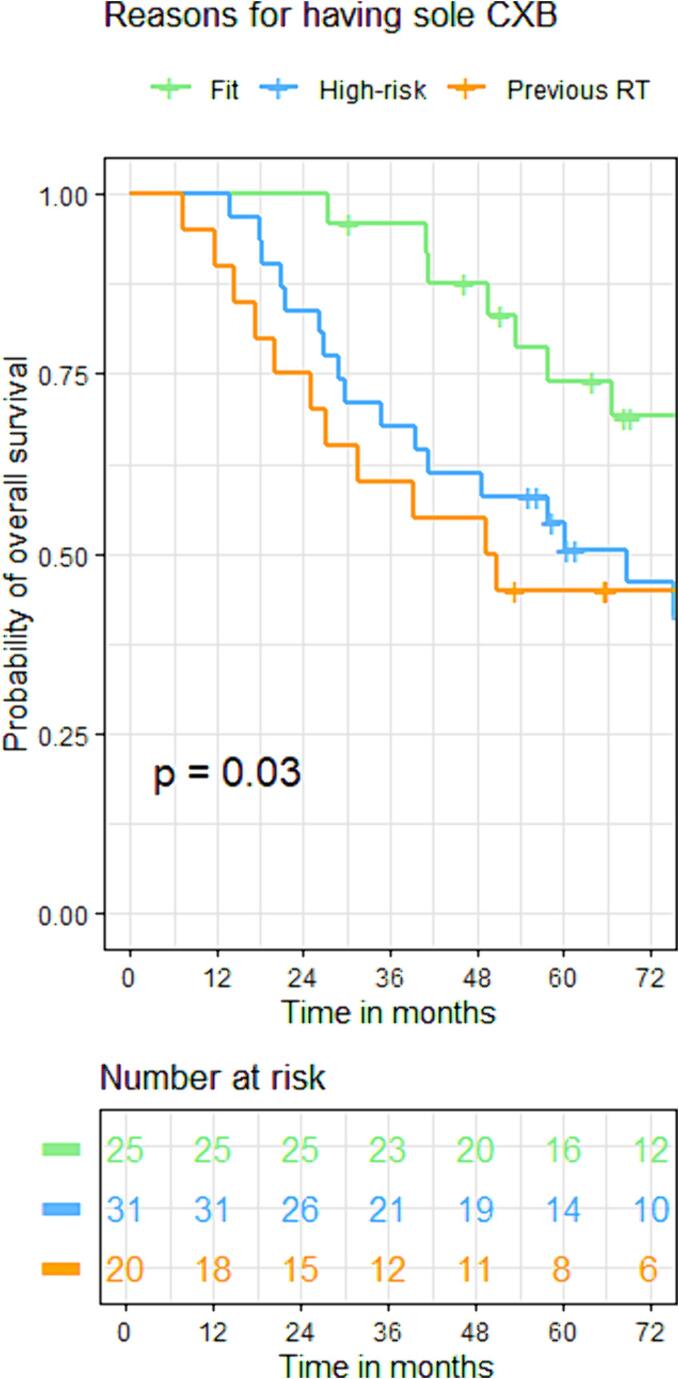
Kaplan-Meier curve for overall survival based on reasons for having CXB alone.

### Predictive factors

Patients with a history of previous pelvic radiotherapy were found to have a lower likelihood of achieving cCR. The patient’s age, performance status, high risk for surgery, and history of prior pelvic radiation were significant predictors for overall survival (Supplementary Table S1).

### Follow-up and salvage treatments

Among the 14 patients who had residual disease following CXB, 5 opted for salvage surgeries: abdominoperineal resections (APER) = 3, low anterior resection (LAR) = 1, and transanal endoscopic microsurgery (TEMS) = 1, with all achieving R0 resections except R1 resection in the TEMS case. Two patients declined surgery and instead received the best supportive care, while the remaining 7 were considered high surgical risk; of these, 2 underwent external beam radiotherapy (EBRT), 1 received palliative chemotherapy, and 4 were given the best supportive care.

Of the 11 patients who experienced local tumour regrowth, 4 underwent APER (all R0 resections), and 1 patient, although fit enough, refused further treatment. The remaining 6 patients were unfit for surgery: 2 received palliative radiotherapy (EBRT and further CXB) and 4 received the best supportive care. Among the two patients who experienced pelvic relapse, one underwent salvage surgery, while the other, who was unfit for surgery, received the best supportive care.

### Post-radiation toxicities

Toxicity profiles were available for 47 out of the 76 patients. The predominant post-treatment symptom was late G1-2 rectal bleeding; observed in 12 patients (26 %). Grade 3 bleeding, necessitating argon beam treatment, occurred in only two patients (4.2 %). Erratic bowel, rectal pain, and urgency suggestive of acute proctitis symptoms (G1-2) manifested in 4 patients (9 %). None of the patients reported symptoms suggestive of impaired anal sphincter function.

## Discussion

In this study, the overall cCR rate was 82 % (range: 70–93) while previous studies that evaluated the efficacy of CXB alone treatment between 1951 and 2006 [13], [14], [32], [33], [34], [35], [36], [37], [38], [39], [40], [41], [42], reported a cure rate of 63–95 % (with 5–20 % rate of residual disease). The local regrowth rate in our study was also comparable to those reported in previous CXB alone studies (18(8–29)% vs 5–28 %). The distant relapse rate was much lower in our study than previous findings (3 % vs 6–13 %), and no patients experienced nodal relapse, whereas one previous study reported a 7 % nodal relapse rate [13]. The 5-year DFS rate was 66(48–78)% vs 66–97 % in the historic series, and the OS rate was 58(44–75)% vs 48–84 %. (Supplementary Table S2) The findings from our study are therefore generally consistent with the results published in these historic series. However, earlier studies utilised various staging systems and methods for assessing response, primarily using DRE and TRUS/ERUS. The development of modern imaging technologies for more accurate staging since those studies were conducted, could potentially introduce bias when directly comparing historical results with those found in our study.

Habr-Gama *et al* demonstrated cCR rates ranging from 57-86 %, locoregional recurrence rates between 20–31 %, and distant relapse rates of 11–26 % in a cohort of 81 patients with early rectal cancers (cT2N0M0) who were treated with standard (50.4 Gy) and extended chemoradiation (54 Gy) [15]. The risk of local recurrence after local excision alone, without adjuvant radiotherapy, for T1 tumours was 4–13 % and 19–29 % for T2 tumours [43], [44]. The heterogeneous nature of patient characteristics in our cohort limits direct comparisons of DFS and OS between our study and these other organ-preserving studies. Despite these constraints, the local control rate reported in our cohort was comparable to those observed with these organ-preserving options, while achieving a lower incidence of distant relapse.

Patients who had T2 tumours, a history of previous pelvic radiotherapy, and who were at high risk for surgery, exhibited lower rates of cCR. However, no statistical significance was observed, which may be attributed to the relatively small cohort sizes. In contrast, patients who were high-risk-for-surgery showed a significantly lower risk of local regrowth than the other groups, thereby achieving a higher rate of DFS. Those patients who had previous radiation exposure for their previous unrelated type of tumour also experienced an increased risk of local tumour relapse, and lower DFS and OS. This may be related to the presence of radioresistant tumour clones and a greater proportion of T2 tumours within this group. Overall, we found no significant differences in outcomes between patients with T1 and T2 tumours, similar to observations in previous studies. However, the results of our statistical analysis may have been influenced by the small sample size of our cohort.

Previous radiation exposure was identified as an adverse predictor for local tumour control and OS, which could be attributed to the potential development of radioresistant tumour clones [45]. Moreover, in this group, CXB alone was used in slightly higher-risk settings (such as T2 tumours) where a combination of EBRT and CXB would probably have been recommended if previous radiation had not been administered to this area.

No serious adverse effects were reported in this study, with comparable rates of self-limiting radiation toxicities, similar to other studies [35], [46]. No patients reported symptoms indicative of impaired anal sphincter function, a common post-treatment side effect of TAE and (chemo)radiation [17], [47].

This study has several limitations. The data primarily relied on a database that was designed to register details such as primary treatment, toxicities, and surgical information. Unfortunately, some information, particularly long-term outcomes for patients who underwent salvage therapies following CXB failures at certain centres, was not recorded. These patients were returned to the care of colorectal surgeons who did not participate in the project, limiting our ability to thoroughly assess the feasibility of salvage treatment and the long-term outcomes for these patients. Moreover, the relatively small cohort size and uneven distribution of patients between sub-groups might have impacted the statistical comparison of outcomes. Additionally, the considerable heterogeneity among patient factors within our cohort may have introduced potential bias when assessing the results of sole CXB and comparing these with outcomes from other treatment options. Therefore, we recommend conducting prospective studies that adequately control for patient and tumour confounding factors to provide a more robust understanding of the role of sole CXB treatment compared to various other organ-preserving treatment options.

## Conclusions

Our study confirmed a high rate of cCR, satisfactory local tumour control, and disease-free survival in a modern cohort of early rectal cancer patients (cT1/cT2, cN0, cM0) who received CXB alone. The lower overall survival rate likely reflects the characteristics of the elderly and comorbid cohort in our study population. Inferior oncological outcomes were also observed in patients with a history of prior pelvic radiotherapy. Nevertheless, considering its several advantages over other organ-preserving treatment options, CXB alone can be considered a viable alternative for selected patients.

## CRediT authorship contribution statement

**Ngu Wah Than:** Conceptualization, Methodology, Software, Formal analysis, Investigation, Data curation, Writing – original draft, Visualization. **D. Mark Pritchard:** Conceptualization, Methodology, Writing – review & editing, Data curation, Supervision, Project administration. **David M. Hughes:** Methodology, Software, Writing – review & editing, Data curation. **Kai Shing Yu:** Resources, Data curation. **Helen S. Minnaar:** Resources, Data curation. **Amandeep Dhadda:** Resources, Data curation, Writing – review & editing. **Jamie Mills:** Resources. **Joakim Folkesson:** Resources, Data curation, Writing – review & editing. **Calin Radu:** Resources, Data curation, Writing – review & editing. **C.A. Duckworth:** Supervision. **Helen Wong:** Resources, Data curation. **Muneeb Ul Haq:** Data curation. **Rajaram Sripadam:** Resources, Data curation. **Mark D. Halling-Brown:** Resources, Data curation. **Alexandra J. Stewart:** Resources, Data curation, Writing – review & editing. **Arthur Sun Myint:** Conceptualization, Resources, Data curation, Writing – review & editing, Supervision.

## Declaration of competing interest

The authors declare that they have no known competing financial interests or personal relationships that could have appeared to influence the work reported in this paper.

## References

[1] Network, N.C.C. NCCN Guidelines Version 2.2024 Rectal Cancer. Available from: https://efaidnbmnnnibpcajpcglclefindmkaj/https://www.nccn.org/professionals/physician_gls/pdf/rectal.pdf;2024. [Assessed 31 May 2024].

[2] Excellence, N.I.f.C., Colorectal cancer. NICE guideline [NG151]. 2020. [Assessed 31 May 2024].

[3] van den Berg I., Coebergh van den Braak R.R.J., van Vugt J.L.A. (2021). Actual survival after resection of primary colorectal cancer: results from a prospective multicenter study. World J Surg Oncol.

[4] Couwenberg A.M., Burbach J.P.M., van Grevenstein W.M.U. (2018). Effect of neoadjuvant therapy and rectal surgery on health-related quality of life in patients with rectal cancer during the first 2 years after diagnosis. Clin Colorectal Cancer.

[5] Morris E.J., Taylor E.F., Thomas J.D. (2011). Thirty-day postoperative mortality after colorectal cancer surgery in England. Gut.

[6] Manceau G., Karoui M., Werner A. (2012). Comparative outcomes of rectal cancer surgery between elderly and non-elderly patients: a systematic review. Lancet Oncol.

[7] Morris E.J.A., Whitehouse L.E., Farrell T. (2012). A retrospective observational study examining the characteristics and outcomes of tumours diagnosed within and without of the English NHS Bowel Cancer Screening Programme. Br J Cancer.

[8] Steele R.J., McClements P., Watling C. (2012). Interval cancers in a FOBT-based colorectal cancer population screening programme: implications for stage, gender and tumour site. Gut.

[9] Bowel Cancer Statistics.; Available from: https://www.cancerresearchuk.org/health-professional/cancer-statistics/statistics-by-cancer-type/bowel-cancer#heading-Zero.2018 [Assessed 18 January 2024].

[10] Barnett K., Mercer S.W., Norbury M. (2012). Epidemiology of multimorbidity and implications for health care, research, and medical education: a cross-sectional study. Lancet.

[11] Bach S.P., Gilbert A., Brock K. (2021). Radical surgery versus organ preservation via short-course radiotherapy followed by transanal endoscopic microsurgery for early-stage rectal cancer (TREC): a randomised, open-label feasibility study. Lancet Gastroenterol Hepatol.

[12] Dhadda A., Sun Myint A., Thamphya B. (2021). A multi-centre analysis of adjuvant contact X-ray brachytherapy (CXB) in rectal cancer patients treated with local excision–Preliminary results of the CONTEM1 study. Radiother Oncol.

[13] Gérard J.P., Ayzac L., Coquard R. (1996). Endocavitary irradiation for early rectal carcinomas T1 (T2). A series of 101 patients treated with the Papillon's technique. Int J Radiat Oncol Biol Phys.

[14] Papillon J. (1975). Intracavitary irradiation of early rectal cancer for cure A series of 186 cases. Cancer.

[15] Habr-Gama A., São Julião G.P., Vailati B.B. (2019). Organ preservation in cT2N0 rectal cancer after neoadjuvant chemoradiation therapy: the impact of radiation therapy dose-escalation and consolidation chemotherapy. Ann Surg.

[16] Steinke J, Jordan C, Rossides S, et al., Planned organ preservation for elderly patients with rectal cancer using short course radiotherapy and a contact brachytherapy boost-an International multi-institution analysis. Clin Transl Radiat Oncol, 2023;39. https://doi.org/10.1016/j.ctro.2023.100580. 10.1016/j.ctro.2023.100580 PMC9852541 36686563

[17] Allaix M.E., Rebecchi F., Giaccone C. (2011). Long-term functional results and quality of life after transanal endoscopic microsurgery. Br J Surg.

[18] Dizdarevic E. (2020). Long-term patient-reported outcomes after high-dose chemoradiation therapy for nonsurgical management of distal rectal cancer. Int J Radiat Oncol Biol Phys.

[19] Lamarque P.L., Gros C. (1946). La radiothérapie de contact des cancers du rectum. J Radiol Electrol.

[20] Papillon J., Berard P. (1992). Endocavitary irradiation in the conservative treatment of adenocarcinoma of the low rectum. World J Surg.

[21] Brodsky J.T., Richard G.K., Cohen A.M. (1992). Variables correlated with the risk of lymph node metastasis in early rectal cancer. Cancer.

[22] Saraste D., Gunnarsson U., Janson M. (2013). Predicting lymph node metastases in early rectal cancer. Eur J Cancer.

[23] Appelt A.L., Pløen J., Vogelius I.R. (2013). Radiation dose-response model for locally advanced rectal cancer after preoperative chemoradiation therapy. Int J Radiat Oncol Biol Phys.

[24] Peter Sminia OG, Kristina Viktorsson, Vidhula Ahire, et al. Clinical Radiobiology for Radiation Oncology, in Radiobiology Textbook, S. Baatout, Editor. 2023, Springer: Springer Nature Switzerland AG. p. 237–242.

[25] Berbée, M., et al., The Role of External Beam and Endoluminal Radiation Boosting in Rectal Cancer. Colorectal Cancer, 2019. 8(1): p. CRC07. Available from: https://www.tandfonline.com/doi/pdf/10.2217/crc-2019-0006 [Assessed 30 June 2024].

[26] Arthur Sun Myint, C.D.L.a.J.P.G., The GEC ESTRO Handbook of Brachytherapy. Rectal Cancer, 2014. Available from: https://www.estro.org/library/item/2453/the-gec-estro-handbook-of-brachytherapy---ch.24--rectal-cancer--2nd-edition---2014-[Assessed 31 May 2024].

[27] Gerard J.P., Pascale R., Jean M.A. (1998). Endocavitary radiation therapy. Semin Radiat Oncol.

[28] Rao C, Smith FM, Martin AP, et al., A Cost-Effectiveness Analysis of Contact X-ray Brachytherapy for the Treatment of Patients with Rectal Cancer Following a Partial Response to Chemoradiotherapy. Clinical oncology (Royal College of Radiologists (Great Britain)), 2018. 30(3): p. 166-177. https://doi.org/10.1016/j.clon.2017.11.015. 10.1016/j.clon.2017.11.015 29248311

[29] Eid Y., Alves A., Lubrano J. (2018). Does previous transanal excision for early rectal cancer impair surgical outcomes and pathologic findings of completion total mesorectal excision? Results of a systematic review of the literature. J Visc Surg.

[30] Morino M., Allaix M.E., Arolfo S. (2013). Previous transanal endoscopic microsurgery for rectal cancer represents a risk factor for an increased abdominoperineal resection rate. Surg Endosc.

[31] Than N.W., Pritchard D.M., Duckworth C.A. (2024). Contact X-ray brachytherapy (CXB) as a salvage treatment for rectal cancer patients who developed local tumor re-growth after watch-and-wait approach. J Contemp Brachytherapy.

[32] Christoforidis D., McNally M.P., Jarosek S.L. (2009). Endocavitary contact radiation therapy for ultrasonographically staged T1 N0 and T2 N0 rectal cancer. Br J Surg.

[33] Frost D.B., Wong R., Rao A. (1993). A retrospective comparison of transanal surgery and endocavitary radiation for the treatment of 'early' rectal adenocarcinoma. Arch Surg.

[34] Hull T.L., Lavery I.C., Saxton J.P. (1994). Endocavitary irradiation - an option in select patients with rectal cancer. Dis Colon Rectum.

[35] Maingon P., Guerif S., Darsouni R. (1998). Conservative management of rectal adenocarcinoma by radiotherapy. Int J Radiat Oncol Biol Phys.

[36] Rauch P., Bey P., Peiffert D. (2001). Factors affecting local control and survival after treatment of carcinoma of the rectum by endocavitary radiation: a retrospective study of 97 cases. Int J Radiat Oncol Biol Phys.

[37] Reed W.P., Cataldo P.A., Garb J.L. (1995). The influence of local tumor ulceration on the effectiveness of endocavitary radiation for patients with early rectal carcinoma. Cancer.

[38] Schild S.E., Martenson J.A., Gunderson L.L. (1996). Endocavitaryradiotherapy of rectal cancer. Int J Radiat Oncol Biol Phys.

[39] Sischy B., Hinson E., Wilkinson D. (1988). Definitive radiation therapy for selected cancers of the rectum. Br J Surg.

[40] de Gara C.J., Harpur G., Basrur V. (1995). The value of endorectal ultrasonography in the follow-up of intracavitary radiation treated early rectal cancer. Surg Oncol.

[41] Kovalic J.J. (1988). Endocavitary irradiation for rectal cancer and villous adenomas. Int J Radiat Oncol Biol Phys.

[42] Mahajan A., Shenouda G., Gordon P.H. (1996). Long source-skin distance rectal irradiation technique: a review of results. Radiother Oncol.

[43] Lee W., Lee D., Choi S. (2003). Transanal endoscopic microsurgery and radical surgery for T1 and T2 rectal cancer. Surg Endosc Other Interv Tech.

[44] van Oostendorp S.E., Smits L.J.H., Vroom Y. (2020). Local recurrence after local excision of early rectal cancer: a meta-analysis of completion TME, adjuvant (chemo)radiation, or no additional treatment. Br J Surg.

[45] Fukui R., Saga R., Matsuya Y. (2022). Tumor radioresistance caused by radiation-induced changes of stem-like cell content and sub-lethal damage repair capability. Sci Rep.

[46] Gerard J.P., Roy P., Coquard R. (1996). Combined curative radiation therapy alone in (T1) T2–3 rectal adenocarcinoma: a pilot study of 29 patients. Radiother Oncol.

[47] van der Sande M.E., Hupkens B.J.P., Berbée M. (2019). Impact of radiotherapy on anorectal function in patients with rectal cancer following a watch and wait programme. Radiother Oncol.

