# Supplementary material for: Contact X-ray Brachytherapy as a sole treatment in selected patients with early rectal cancer – Multi-centre study
Source: Clin Transl Radiat Oncol. 2024 Sep 6;49:100851. doi: 10.1016/j.ctro.2024.100851 (PMC11414538; doi:10.1016/j.ctro.2024.100851)
Supplement: Supplementary Data 1 [file mmc1.docx]

Supplementary table 1:Factors predicting the treatment response

| Factor | Residual disease | | Local regrowth | | Disease-free survival | | Overall survival | |
| --- | --- | --- | --- | --- | --- | --- | --- | --- |
|  | OR (95%CI) | P value | OR (95%CI) | P value | HR (95%CI) | P value | HR (95%CI) | P value |
| Age | 1.0(0.9, 1.0) | 0.71 | 1.0(0.9,1.1) | 0.98 | 1.1(0.3,3.8) | 0.90 | 3.3(1.0,10.8) | 0.04 |
| WHO performance status | 1.9(0.5,6.5) | 0.33 | 0.6(0.1,2.4) | 0.45 | 0.5(0.2,1.9) | 0.32 | 2.3(1.2,4.4) | 0.01 |
| Prior pelvic RT  High-risk for surgery | 6.0(1.1,33.9) | 0.04 | 0.6(0.1,2.5) | 0.46 | 1.7(0.6,5.3) | 0.35 | 2.3(1.1,4.9)  2.5(1.1,5.7) | 0.02  0.04 |
| T-stage | 4.1(0.8,19.7) | 0.08 | 0.7(0.2,2.6) | 0.63 | 1.2(0.4,3.1) | 0.75 | 1.6(0.8,3.2) | 0.15 |
| Distance from anal verge | 1.1(0.3,4.0) | 0.91 | 0.3(0.1,2.3) | 0.22 | 0.6(0.1,2.9) | 0.55 | 1.5(0.8,3.1) | 0.22 |
| Tumour size | 0.9(0.5,1.8) | 0.84 | 1.4(0.7,2.9) | 0.31 | 0.7(0.1,5.8) | 0.78 | 0.7(0.2,2.2) | 0.51 |
| Flat/Ulcerated | 3.8(0.6,22.2) | 0.15 | 4.3(0.6,31.1) | 0.15 | 6.2(1.0,38.0) | 0.05 | 1.1(0.4,3.0) | 0.84 |
| CXB total dose | 1.5(0.4,6.0) | 0.57 | 1.2(0.29,4.84) | 0.82 | 1.1(0.4,3.4) | 0.85 | 1.0(0.5,1.9) | 0.94 |
