# Supplementary material for: Contact X-ray Brachytherapy as a sole treatment in selected patients with early rectal cancer – Multi-centre study
Source: Clin Transl Radiat Oncol. 2024 Sep 6;49:100851. doi: 10.1016/j.ctro.2024.100851 (PMC11414538; doi:10.1016/j.ctro.2024.100851)
Supplement: Supplementary Data 2 [file mmc2.docx]

**Supplementary table2: Previous studies on the outcomes of sole CXB treatment**

| Study  (Study period) | Patient number | Median age  (IQR) | Stage | Tumour size (cm)  (Median/Median) | Staging Method | Total dose (Gy) | Median Follow-up  (months) | RD | LR | NR | DR | Cure rate | 5-year  DFS (%) | 5-year  OS (%) | Toxicities |
| --- | --- | --- | --- | --- | --- | --- | --- | --- | --- | --- | --- | --- | --- | --- | --- |
| Christoforidis et al.  (1986-2006) | 77 | 74  (38-104) | T1=40  T2=37 | 2·5  (1–4·5) | ERUS | 60-190 | 69 | 4/77  (5%) | 15/73  (21%) | NA | 5/77  (6%) | 56/77  (73%) | 74-87 | 66 | Proctitis (13%)  G1/2 RB (12%)  G3 RB (9%) |
| De Gara et al.  (1989-1991) | 30 | 65  (37-78) | T1-2 | NA | ERUS | 90-150 | 24 | 6/30  (20%) | NA | NA | NA | 24/30  (80%) | NA | NA | NA |
| Frost et al.  (1984-1989) | 38 | range  52-80 | Early | 2.5 | NA | 100-120 | 39 | NA | 8/38  (21%) | NA | NA | 30/38  (79%) | 97  Absolute | 84  Absolute | NA |
| Gerard et al.  (1977-1993) | 101  Combined with ^192^Ir=28 | 73  range  (35-91) | T1=65  T2=36 | 2.5 | TRUS | 60-125 | 61 | 0 | 8/101  (8%) | 7/101  (7%) | 6/101  (6%) | NA | 94 | 83 | Proctitis (15%)  RB (46%) |
| Hull et al.  (1973-1992) | 126 | 66  (38-99) | NA | NA | NA | 60-180 | 51 | NA | 27/126  (21%) | NA | 10  (8%) | 89/126  (71%) | NA | NA | NA |
| Kovalic et at.  (1978-1987) | 32 | 71  (53-98) | NA |  | DRE | 60-130 | 35 | NA | 24% | NA | NA | NA | 74  (3-year) | NA | RB (60%)  Proctitis (17%) |
| Mahajan et al.  (1986-1993) | 14 | 73  (46-86) | NA | ≤3 | DRE | 60-135 | 24 | 2/14  (14%) | 2/12  (17%) | NA | 1/14  (7%) | 9/14  (64%) | 71  Absolute | 51  Absolute | Proctitis (29%)  RB (50%) |
| Maingon et al.  (1975-1995) | 93 | 71  Range  (25-92) | Dijon  T1-T2 | >3 | TRUS | 90-120 | 60 | NA | 13-28% | NA | NA | NA | 66 | 48-70 | Proctitis (37%)  RB (3%) |
| Papillon et al.  (1951-1972) | 186 | NA | Early | <5 | DRE | 100-150 | 60 | NA | 7.5% | NA | 9% | 91% | NA | 78 | NA |
| Reed et al.  (1984-1991) | 32 | 73  (52-78) | Duke A | 2.1  (0.5-4.0) | DRE+  TRUS | 75-120 | 43 | NA | 8/32  (25%) | NA | 4/32  (13%) | 20/32  (63%) | NA | NA | RB (41%)  Proctitis (28%) |
| Schild et al.  (1987-1994) | 20 | 75 | Early | NA | TRUS | 20-155 | 55 | NA | 2/20  (10%) | NA | NA | 18/20  (90%) | 89 | 75 | RB (5%)  Pain (5%) |
| Sischy et al.  (1973-1987) | 192 | NA | Duke  A-B | NA | DRE | 100-120 | 60 | NA | 9/192  (5%) | NA | NA | 183/192  (95%) | NA | NA | NA |
| Tanum et al.  (1989-1995) | 12 | 78  (57-90) | Early | 2.3 | TRUS | 120-200 | 46 | 1/12  (8%) |  | NA | 1/12  (8%) | 10/12  (84%) | NA | NA | RB  (several patients) |
| IQR: inter-quartile range, RD: residual disease, LR: local regrowth, NR: nodal relapse, DR: distant relapse, DFS: disease-free survival, OS: overall survival, RB: rectal bleeding | | | | | | | | | | | | | | | |
